# Prediction of Neurodevelopmental Disorders Based on De Novo Coding Variation

**DOI:** 10.1007/s10803-022-05586-z

**Published:** 2022-05-20

**Authors:** Julie C. Chow, Fereydoun Hormozdiari

**Affiliations:** 1 UC Davis Genome Center, University of California, Davis, CA 95616 USA; 2 MIND Institute, University of California, Davis, 95817 USA; 3 Biochemistry and Molecular Medicine, University of California, Davis, 95616 USA

**Keywords:** De novo mutation, Early prediction, Neural network, Likely gene-disruptive, Missense

## Abstract

The early detection of neurodevelopmental disorders (NDDs) can significantly improve patient outcomes. The differential burden of non-synonymous de novo mutation among NDD cases and controls indicates that de novo coding variation can be used to identify a subset of samples that will likely display an NDD phenotype. Thus, we have developed an approach for the accurate prediction of NDDs with very low false positive rate (FPR) using de novo coding variation for a small subset of cases. We use a shallow neural network that integrates de novo likely gene-disruptive and missense variants, measures of gene constraint, and conservation information to predict a small subset of NDD cases at very low FPR and prioritizes NDD risk genes for future clinical study.

**Supplementary Information:**

The online version contains supplementary material available at 10.1007/s10803-022-05586-z.

## Introduction

Neurodevelopmental disorders (NDDs), such as autism spectrum disorder (ASD), epilepsy, intellectual disability (ID), and developmental disability (DD) are complex disorders characterized by impairment in cognition, learning, and motor skills. From twin and family studies, it has become apparent that NDDs possess a strong genetic component (Freitag, 2007; Gejman et al., 2010). Estimates of heritability for various NDDs have ranged from 0.3 to 0.9, with heritability estimated to be greater than 0.5 for both ASD and ID (Flint, 2001; Kaufman et al., 2010; Tick et al., 2016). The evident contribution of genetic factors to NDD diagnoses has provided reason for routine prenatal whole exome or genome sequencing to identify potentially deleterious genetic variations (Soden et al., 2014; Tărlungeanu & Novarino, 2018). In particular, whole exome sequencing has proved a useful tool to identify, at a low cost, coding variants in genes that are highly intolerant to mutation and play important roles in typical neurodevelopment (Srivastava et al., 2019).

The identification and prioritization of NDD risk genes is important for the discovery of underlying biological mechanisms that are perturbed in NDDs (Cardoso et al., 2019). Previous studies have identified many monogenic forms of NDDs and revealed the multifactorial and polygenic nature of most NDD diagnoses (De Felice et al., 2015; Niemi et al., 2018; Sztainberg & Zoghbi, 2016). In particular, rare de novo mutations that are observed in genes in NDD cases at a significantly higher rate than expected relative to unaffected controls have pinpointed many candidate NDD genes, with more than one thousand genes estimated to be NDD risk genes (De Rubeis et al., 2014; Heyne et al., 2018; Iossifov et al., 2012; Kaplanis et al., 2020; McRae et al., 2017; O’Roak et al., 2012a, b; Sanders et al., 2012; Satterstrom et al., 2020; Wilfert et al., 2017).

De novo mutations are a class of rare genetic variation in which variants, that are not observed in parental genomes, exist in offspring due to mutagenesis in germ cells or errors in replication or recombination (Acuna-Hidalgo et al., 2016). De novo mutations may exist as single nucleotide variants, insertions and deletions (indels), and copy number variants. Because de novo mutations are not inherited, highly penetrant mutations can arise in genes that are critical to neurodevelopment and likely under purifying selection (Iossifov et al., 2012; Uddin et al., 2014). In fact, individuals affected by NDDs experience a greater burden of non-synonymous de novo mutation compared to unaffected controls (Coe et al., 2019; Wilfert et al., 2017). Study of ASD simplex families from the Simons Simplex Collection (SSC) has found that de novo likely gene disruptive (LGD) mutations occur at a nearly twofold increased rate in affected cases (0.21) relative to controls (0.12), as well as displaying an increased rate of missense mutation (Iossifov et al., 2014). Furthermore, the study of genetic modules impacted by these de novo mutations has pinpointed several biological processes relevant to NDD etiology, such as chromatin remodeling, the Wnt pathway, synaptic transmission, and the long-term potentiation pathway (Chow et al., 2019; Kwan et al., 2016; O’Roak et al., 2012a, b; Wilfert et al., 2017).

The benefits associated with successful early prediction of NDDs include the improved ability of parents to make informed decisions about potential early application of treatments (Boivin et al., 2015; Cioni et al., 2016; Corsello, 2005). It is important to note that most NDDs cases cannot be predicted using de novo coding variation alone; the exome constitutes 1–2% of the human genome and the majority of NDD-associated variants are likely to reside in non-coding regions involved in the regulation of gene expression (Short et al., 2018; Turner & Eichler, 2019). Currently, it is estimated that only ~ 10% of ASD cases and ~ 20–30% of ID/DD cases have de novo LGD variants, and the rate of such variants in the general population is significantly lower (Wang et al., 2021). The genetically and phenotypically heterogeneous nature of NDDs indicates that many factors, including common and non-coding genetic variants and non-genetic factors, account for a large fraction of diagnoses, further complicating our ability for the early prediction of these disorders. However, it is possible to confidently predict a subset of individuals who will likely develop NDDs due to de novo coding variation in the form of non-synonymous de novo mutations. Despite the polygenic nature of NDDs and the multitude of potential genetic or environmental causes, focusing specifically on un-inherited, de novo mutations that disrupt protein coding sequence permits early prediction for a small fraction of cases with very low false positive rates.

The early prediction of NDDs requires a very low false positive rate (FPR) due to potential negative consequences, such as the costs associated with early intervention treatments, that may result from false positive prediction. The minimization of the FPR is clinically most relevant in genetic counseling settings for parents with suspected or confirmed familial risk for NDDs and to aid in the decision to begin early intervention treatments in young children. Early diagnosis of NDDs via a combination of behavioral and motor assessments, imaging, and genetic testing followed by early prediction methods can greatly benefit patient outcomes and lead to timely, appropriate treatment (Hadders-Algra, 2021). Previously, a method for the early prediction of complex disorders, Odin, used de novo LGD variants observed in cases and controls and co-expression data to identify cases at very low FPR (Huynh & Hormozdiari, 2018). The shallow neural net (SNN) with novel objective function introduced here incorporates LGD de novo mutation, constraint, and conservation data to achieve a higher (> 0.30129) true positive rate (TPR) at very low FPR (< 0.01) in comparison to traditional classification models such as random forest (RF), support-vector machine (SVM), and logistic regression (LR). Furthermore, the proposed SNN model achieves similar PR-AUC and ROC-AUC to other machine learning approaches. An ensemble model that averages predictions among the SNN, RF, SVM, and LR models is able to achieve a slightly increased TPR at FPR < 0.01 and comparable PR-AUC. Additionally, the SNN is able to rank genes according to their relative importance in NDDs given LGD or missense de novo variation, prioritizing candidate NDD genes.

## Methods

### Objective

The main objective is to investigate the potential of using machine learning approaches for early prediction of NDDs using de novo coding genetic variants in a subset of cases. More formally, we are interested in utilizing de novo coding variants in maximizing the fraction of affected NDD cases accurately predicted when limiting the FPR to virtually zero.

#### Data Collection and Preprocessing

To distinguish NDD cases from unaffected controls using de novo coding variation, de novo likely gene-disruptive (LGD) and missense variants were retrieved from denovo-db (version 1.6.1) (Turner et al., 2017, p.). These data consisted of 9962 individuals with primary phenotypes of autism spectrum disorder (ASD), intellectual disability, and developmental disability and 2245 controls, of which 6509 cases and 1251 controls possess non-synonymous coding de novo mutation (Supplementary Table 1). In total, the 7760 samples possessed 1974 LGD (cases: 1715; controls: 259) and 10,777 (cases: 9073; controls: 1704) missense de novo coding mutations. *PrimateAI* scores were used to quantify the pathogenicity of missense variants, in which position-specific scores were calculated for each missense variant while incorporating conservation, solvent accessibility, and secondary structure data to yield predictions of deleteriousness (Sundaram et al., 2018). Probability of loss-of-function intolerance (pLI) and loss-of-function observed/expected upper bound fraction (LOEUF) scores from the gnomAD browser (v2.1.1), Residual Variation Intolerance (RVIS) scores based on ExAC v2 release 2.0 (March 15, 2017 version), and phastCons element scores were also used as features (Karczewski et al., 2020; Petrovski et al., 2013; Siepel et al., 2005).

**Fig. 1:**
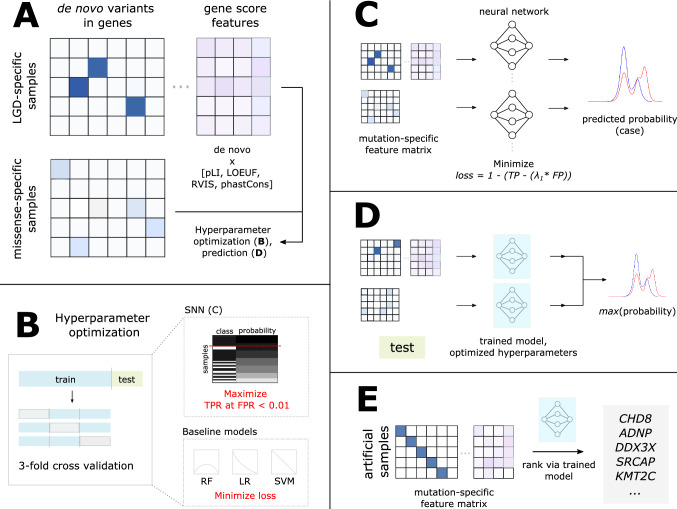
Methods overview. **A** De novo LGD and missense variants from probands with NDDs and controls were retrieved from denovo-db and arranged into feature matrices. Constraint and conservation information, in the forms of pLI, LOEUF, RVIS, and average phastCons element conservation scores were incorporated as gene score features (Karczewski et al., 2020; Petrovski et al., 2013; Siepel et al., 2005) (Additional File 1). **B** To perform hyperparameter optimization and model training, samples were divided into training (75%) and testing (25%) sets. Hyperparameter optimization occurs via threefold cross validation on the partitioned training set. For the SNN model (**C**), performance is measured as the TPR at FPR < 0.01, which is calculated by determining the number of cases (class: black) with predicted probability greater than that of any control (class: white) in the validation fold. For baseline models, consisting of the RF, LR, and SVM classifiers, respective loss functions are minimized. **C** The SNN consists of a single hidden layer and a loss function that seeks to minimize the product of predicted FP and a parameter ${\lambda }_{1}$, subtracted from the TP. **D** During the prediction phase, using the model trained with optimized hyperparameters, a prediction is made on the withheld testing set. For samples that simultaneously have both LGD and missense variation, two separate probabilities are retrieved from LGD- and missense-specific models for a given individual, and the maximum predicted probability is returned per individual. **E** For ranking genes based on their importance to NDDs, artificial samples are generated such that each artificial sample has a single de novo variant in a unique gene, using either LGD or missense variation, separately. Application of the prediction phase (**D**) on artificial samples yielded a ranking of the relative importance of a gene to NDDs determined via de novo coding variation

LGD-specific and missense-specific feature matrices were generated, in which rows represent individuals with LGD or missense variation from denovo-db and columns represent genes containing de novo mutations (Fig. 1A, Additional File 1).

#### Model Architecture Development and Hyperparameter Tuning

Separate models were trained for de novo LGD variation and missense variation, referred to as SNN LGD-specific and missense-specific models. Each variation-specific SNN consists of two phases, a hyperparameter optimization phase and a prediction phase. After splitting all samples into training (75%) and testing (25%) sets, the hyperparameter optimization phase is applied to the training set, choosing optimal hyperparameters within a selected search space (Fig. 1B, Supplementary Table 2, Additional File 1). The purpose of the hyperparameter optimization phase for the SNN is to select a set of hyperparameters that yield the largest TPR at very low FPR on the training set to use during the prediction phase. Similarly, RF (sklearn.ensemble.RandomForestClassifier), SVM (sklearn.svm.LinearSVC), and LR (sklearn.linear_model.LogisticRegression) classifiers, hereon referred to as baseline models, are individually subjected to hyperparameter optimization and prediction phases. To allow direct comparison of each model’s performance, identical training/testing splits are provided to SNN and baseline models. The performance of SNN and baseline models are additionally compared to the TPR and FPR of the following heuristics, in which an individual is classified as a case if the individual has an LGD mutation in: (1) any gene with a (i) SFARI score of 1 (high confidence ASD gene) or (ii) SFARI score of 1 or 2 (strong candidate ASD gene) (https://gene.sfari.org/database/gene-scoring/), (2) any gene identified by SPARK as a (i) prioritized or (ii) risk gene, and (3) any gene with i) pLI >  = 0.90 or (ii) LOEUF < 0.35 (Additional File 1).

In the hyperparameter optimization and prediction phases (Fig. 1C),

1 $$loss=1-(TP- {(\lambda }_{1}*FP))$$

is used as a custom loss function (Eq. 1) for the SNNs, in which the objective is to minimize the product of the number of false positives (FP) and the hyperparameter ${\lambda }_{1}$ subtracted from the true positives (TP). The value of ${\lambda }_{1}$ is selected during the hyperparameter optimization phase. The SNN architecture consists of an input layer, a hidden layer with ReLU activation and an optimized number of neurons, and an output layer with sigmoid activation and L2 regularization with an optimized regularization parameter ${\lambda }_{2}$. The SNN uses the Adam optimization algorithm.

To return a prediction that incorporates both LGD and missense variation for individuals who possess both types of variants simultaneously (referred to as a ‘combined’ prediction), predictions are retrieved from the separately trained LGD- and missense-specific models for SNN and baseline models. For a given sample with both LGD and missense variation, the maximum predicted probability from the two separately trained variation-specific models is returned as the predicted probability of being a case primarily due to de novo coding variation (Fig. 1D). By using the maximum predicted probability, the model is trained to learn the class of an individual given their de novo mutation that is predicted to have the largest deleterious effect.

The average performance of a model over 100 independent training and testing splits is measured by determining the average TPR at FPR < 0.01, ROC-AUC, and PR-AUC for LGD-specific, missense-specific, and combined predictions for the SNN approach using the custom loss function, three baseline models, an ensemble model, and an ensemble model excluding SNN predictions (Additional File 1). To demonstrate the importance of gene score features and PrimateAI scores to increased TPR at FPR < 0.01, SNN and baseline models were trivially trained on one-hot encoded feature matrices indicating only the presence or absence (denoted as 1 or 0, respectively) of de novo LGD or missense mutation, and performance metrics were returned. To additionally assess the performance of the missense-specific model using only deleterious missense variation with PrimateAI scores ≥ 0.803 (as described in Sundaram et al., 2018), the missense-specific model (i) was trained using only samples with deleterious missense variation (PrimateAI ≥ 0.803) without discarding any samples, or (ii) was executed while excluding samples without deleterious missense mutation from training and testing sets.

#### NDD Gene Ranking

To rank genes according to their relative importance to NDDs using de novo coding variation in the form of de novo LGD mutations or missense mutations, two sets of artificial samples (LGD- and missense-specific) were created. The artificial samples each contain a single LGD (or missense) variant in a unique gene in the human genome (Fig. 1E). The probability of being a case is predicted for each of these artificial samples using the previously trained SNN LGD- or missense-specific models. For every artificial sample and its corresponding gene containing a de novo LGD (or missense) variant, the predicted probability indicates the relative importance of the gene to NDD risk from de novo coding variation. Enrichment of de novo LGD and missense mutation in NDD cases relative to controls was assessed (Additional File 1).

## Results

To identify, at very low FPRs, a subset of affected NDD cases using rare coding variation consisting of de novo LGD and missense variation, LGD- and missense-specific feature matrices indicating the presence of de novo variation within genes were constructed (Fig. 1A). Additional features incorporating constraint and conservation data were used to improve classification of NDD cases using LGD variation. The ability of SNNs (Fig. 1C) to classify NDD cases at very low FPRs were compared to various classifiers, including RF, SVM, and LR (baseline models), in addition to three heuristics.

### De Novo LGD Mutations Distinguish a Subset of NDD Cases from Controls with Low FPR

**Fig. 2:**
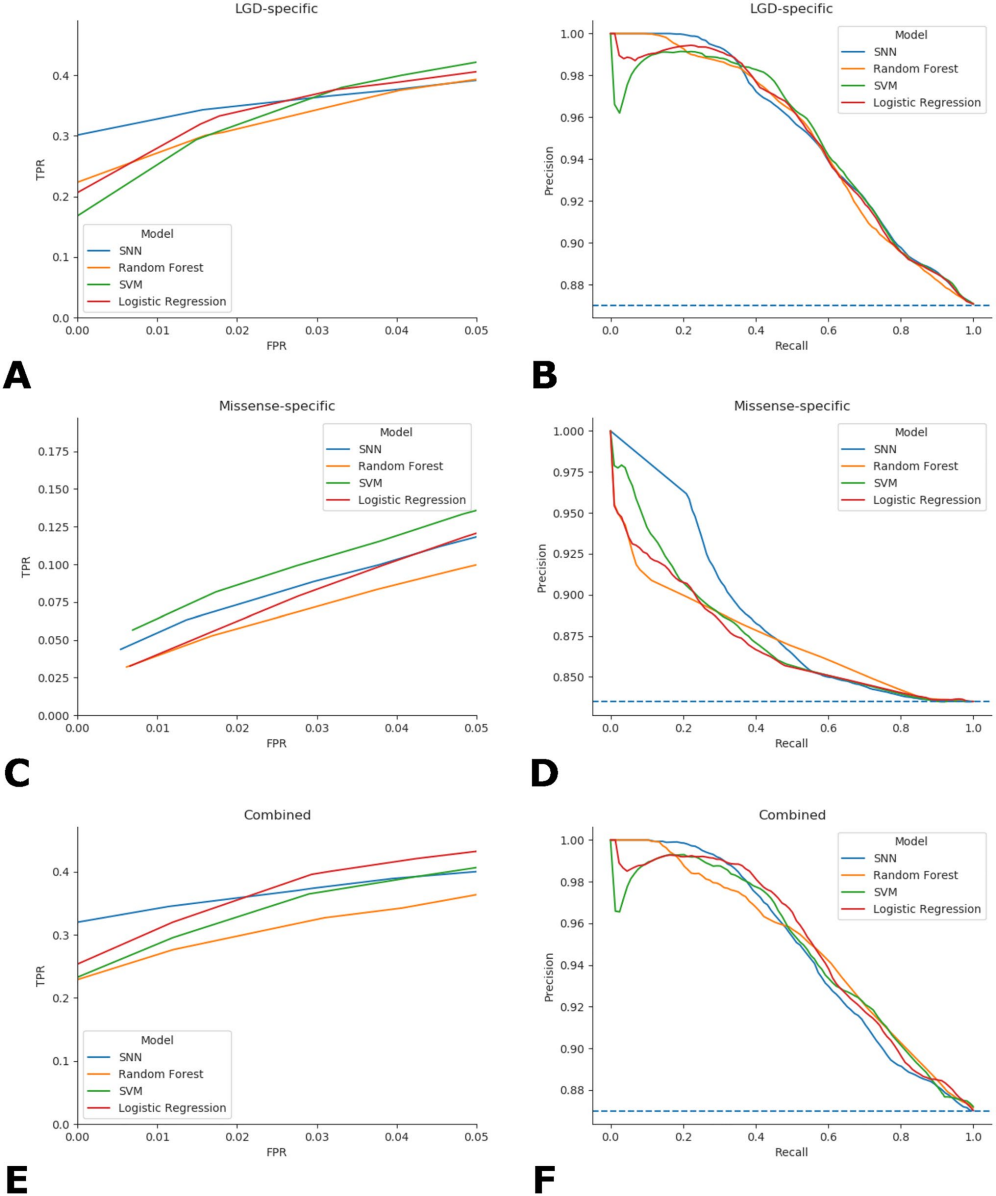
Receiver operating characteristic and PR curves for LGD- and missense-specific and combined predictions for SNN and baseline (RF, SVM, and LR) models. Random classification is displayed as a dashed blue line in all PR curves. Models trained on LGD-specific variation feature matrices additionally use constraint and conservation gene score information, whereas models provided with missense-specific feature matrices do not use gene score information. For LGD-specific features, the SNN achieves greater TPR at low FPR < 0.01 compared to baseline models, a trend which is evident even at FPR < 0.05 (**A**), and the SNN achieves comparable precision at lower recall compared to baseline models (**B**). Models trained on missense-specific variation alone are poor predictors of NDD status; SNN and baseline models show similar TPR at FPR < 0.05, with the SNN achieving slightly higher rates at low FPR (**C**). The SNN displays comparable precision at low recall thresholds when trained on missense-specific variation (**D**). **E** For combined prediction for samples with both missense and LGD variation, the proportion of cases captured at FPR < 0.01 is largest for the SNN, and similar precision at low recall is observed for the SNN compared to baseline models (**F**)

At very low FPRs (FPR < 0.01), an SNN trained on an LGD-specific feature matrix captures 30.1% of NDD cases possessing any de novo LGD coding variation. In comparison to baseline models, the SNN trained on an LGD-specific feature matrix is able to identify 5.29–10.25% [95% confidence interval (CI)] more NDD cases at FPR < 0.01 than the RF classifier, and more than 5.73–17.26% (95% CI) NDD cases than SVM or LR models (Fig. 2, Table 1, Supplementary Fig. 1). To measure the extent to which the SNN and other models achieve increased TPR at FPR < 0.01 compared to a randomized model, a z-score was also calculated (Additional File 1, Table 1).

**Table 1:** Average TPR at FPR < 0.01, ROC-AUC, and PR-AUC for LGD-specific, missense-specific, and combined SNN, baseline, ensemble models, and randomized predictions

Input features	Model	TPR at FPR < 0.01 (95% CI); z-score	ROC-AUC (95% CI); z-score	PR-AUC (95% CI); z-score
LGD-specific	SNN	0.30129 (0.2906, 0.3124); 4.93244	0.72785 (0.7227, 0.7326); 4.01329	0.95050 (0.949, 0.9519); 5.86600
	Random forest	0.22342 (0.2099, 0.2377); 2.83170	0.71997 (0.7154, 0.7244); 3.95991	0.94866 (0.9472, 0.95); 5.81660
	SVM	0.16790 (0.1398, 0.1962); 1.04685	**0.73199** (0.7278, 0.7365); 4.18017	0.94825 (0.9463, 0.9498); 5.33855
	Logistic regression	0.20632 (0.18, 0.2333); 1.34869	0.72695 (0.7222, 0.7317); 4.06566	0.94877 (0.9471, 0.9504); 5.58760
	Ensemble	**0.30715** (0.2965, 0.3174); 5.08163	0.73037 (0.7261, 0.7347); 4.14049	**0.95176** (0.9504, 0.953); 6.08741
	Ensemble—SNN	0.23347 (0.2213, 0.2453); 3.33032	0.72823 (0.724, 0.7325); 4.10213	0.95023 (0.9488, 0.9515)); 6.00325
	Randomized	0.01660 (0.0135, 0.0202)	0.50627 (0.4963, 0.5164)	0.8698
Missense-specific	SNN	0.02334 (0.0199, 0.0267); 1.09477	0.54378 (0.5391, 0.5483); 1.23832	**0.88139** (0.878, 0.885); 2.40309
	Random forest	0.01279 (0.0109, 0.0151); 0.78867	0.53086 (0.5287, 0.533); 1.17197	0.87220 (0.8705, 0.8738); 3.97519
	SVM	**0.02610** (0.022, 0.0301); 1.09631	0.55910 (0.5564, 0.5618); 2.22556	0.87486 (0.8737, 0.876); 5.88837
	Logistic regression	0.01214 (0.0101, 0.0144); 0.72456	0.55810 (0.5551, 0.5609); 2.13551	0.87071 (0.8694, 0.872); 4.82097
	Ensemble	0.02530 (0.022, 0.0288); 1.18239	**0.56006** (0.5571, 0.5631); 2.18983	0.87374 (0.8726, 0.8749); 5.71154
	Ensemble—SNN	0.02386 (0.0205, 0.0272); 1.13687	0.55915 (0.5564, 0.5619); 2.18614	0.87383 (0.8726, 0.8751); 5.69270
	Randomized	0.00406 (0.0033, 0.0048)	0.50304 (0.4991, 0.5071)	0.8350
Combined	SNN	0.31985 (0.3038, 0.3348); 3.55285	0.71422 (0.7071, 0.7215); 2.93749	0.94685 (0.9445, 0.949); 3.95676
	Random forest	0.22892 (0.2129, 0.2456); 2.39793	0.71830 (0.7121, 0.7246); 3.15223	0.94740 (0.9453, 0.9494); 4.02305
	SVM	0.23267 (0.2058, 0.2598); 1.41386	0.72803 (0.7211, 0.7346); 3.21778	0.94620 (0.9437, 0.9486); 3.67270
	Logistic regression	0.25347 (0.226, 0.2837); 1.48639	0.73280 (0.7269, 0.7389); 3.34153	0.94874 (0.9466, 0.951); 4.05063
	Ensemble	**0.33567** (0.3216, 0.3508); 3.87773	**0.74128** (0.7345, 0.7481); 3.42116	**0.95215** (0.9501, 0.9541); 4.35673
	Ensemble—SNN	0.23961 (0.2249, 0.2549); 2.63914	0.73737 (0.7302, 0.7447); 3.30974	0.94899 (0.9468, 0.951); 4.09443
	Randomized	0.02898 (0.0224, 0.036)	0.53177 (0.5218, 0.5409)	0.8701

An ensemble model generated only from the predictions of baseline models while excluding SNN predictions is referred to as ‘Ensemble—SNN’. To generate randomized predictions, probabilities drawn from a uniform distribution were randomly assigned to samples. Average performance metrics are measured over 100 independent iterations of randomized training/testing splits on the testing set, in which the same training/testing partition is provided to all models at each iteration. Confidence intervals (95% CI) are indicated in parentheses, followed by a z-score quantifying the deviance from the mean performance metric of a certain model and the randomized model (Additional File 1). The PR-AUC values associated with randomized predictions were calculated by dividing the number of cases in a testing set by the total number of samples within the testing set. The largest average TPR at FPR < 0.01, ROC-AUC, and PR-AUC values are bolded for LGD-specific, missense-specific, and combined models

For the SNN, ROC-AUC and PR-AUC values of 0.72785 (0.7227–0.7326, 95% CI) and 0.9505 (0.9490 to 0.9519, 95% CI), respectively, were observed (Table 1). Observed PR-AUC values were comparable among the SNN and baseline models in their deviance from the randomized model, displaying similar z-scores. Note that due to the large number of cases in proportion to controls in available datasets, PR-AUC values for SNN and baseline models are significantly inflated; the random assignment model has an PR-AUC of over 0.85.

The inclusion of gene score features derived from pLI, LOEUF, RVIS, and phastCons element scores improves upon an SNN trivially trained only on LGD mutations (TPR at FPR < 0.01 = 0.24532, ROC-AUC = 0.66696, PR-AUC = 0.93597) (Supplementary Table 3, Supplementary Fig. 2). In addition, baseline and SNN models yield similar performance metrics when trivially trained on only LGD mutations, indicating. that the inclusion of gene constraint and conservation information is important to accurate classification of NDD cases using de novo LGD mutations (Supplementary Table 3).

Compared to the TPR and FPR of the three previously described heuristics, in which a sample was classified as a case if the sample possessed an LGD mutation in a set of prioritized genes, decreased TPR at low FPR thresholds in comparison to the SNN was observed for each heuristic (Supplementary Table 4, Supplementary Fig. 3). No heuristic achieved similar TPR values greater than 0.30 at FPR less than 0.01.

### Integration of Missense and LGD-Specific Models Improves Prediction on Individuals with Both De Novo Missense and LGD Variants

To assess the ability of de novo missense mutations to distinguish NDD cases from unaffected controls, de novo missense variants from individuals with at least one missense variant were retrieved, consisting of 6947 samples possessing a total of 10,777 missense mutations. SNN and baseline models trained on missense variation capture less than 2.6% of NDD cases at FPR < 0.01 (Fig. 2, Table 1), indicating that accurate prediction of NDDs using only missense de novo variants is an extremely challenging problem. Slightly increased TPR at FPR < 0.01 is observed when the missense-specific model is trained only on deleterious missense variation without removing any samples from training and testing; excluding samples without deleterious missense variation from training and testing yields 2242 samples (2257 cases; 248 controls) with 2,505 deleterious missense variants and increased TPR at FPR < 0.01 (Supplementary Table 5).

For samples possessing both de novo missense and LGD variants, accurate prediction of NDD cases at low FPR can be improved by taking the maximum predicted probability from two models trained separately on only missense or LGD variation, referred to as a ‘combined’ prediction (Fig. 2, Table 1). Combined prediction on samples with both missense and LGD variation captures an increased fraction of cases. For example, compared to the LGD-specific SNN, an SNN using combined prediction is able to detect at most 4.22% more affected cases at FPR < 0.01 (95% CI). TPR at FPR < 0.01—associated z-scores for the SNN are greater by 1.41- 2.51 than values observed for baseline models using combined predictions.

### Ensemble Prediction Yields Increased TPR at Very Low FPRs Compared to Separately Trained SNN and Baseline Models

An ensemble prediction was generated by returning the average predicted probability from the SNN, RF, SVM, and LR models for a given sample in the testing set. Compared to SNN and baseline models for LGD-specific, missense-specific, and combined models, the ensemble model consistently yields a larger TPR at low FPR values (Supplementary Fig. 4, Table 1). The predictive contribution of the SNN to the ensemble model is more substantial than that of the baseline models. For example, the TPR at FPR < 0.01 is greater for LGD-specific and combined prediction SNNs than ensemble models that exclude SNN predictions, referred to as ‘Ensemble—SNN’ (Table 1, Supplementary Fig. 4). Additionally, for LGD-specific and combined predictions, there is no overlap of 95% CIs between SNN and Ensemble—SNN models. From the ensemble prediction's constituent models, the SNN performs most similarly to the full ensemble method, differing by 0.586% and 1.582% in TPR at FPR < 0.01 given LGD-specific and combined predictions, respectively. In addition, the ensemble model achieves a slightly higher average PR-AUC, as evidenced by an increased z-score, than any individual SNN or baseline model for corresponding LGD-specific (0.95176) or combined predictions (0.95215) (Table 1).

### Integration of Constraint, Conservation, and De Novo Mutation Data Permit NDD Gene Prioritization

Training of SNNs (Fig. 1C) on variation-specific feature matrices enables NDD risk gene ranking according to the effect of de novo missense and LGD mutations within specific genes (Fig. 1E). For example, using only LGD variants during SNN training reveals genes that are sensitive to LGD mutations and play important roles in typical neurodevelopment. Gene rankings and associated SFARI Gene scores are displayed in Supplementary Table 6 in descending order according to their relative importance to NDD risk.

**Fig. 3:**
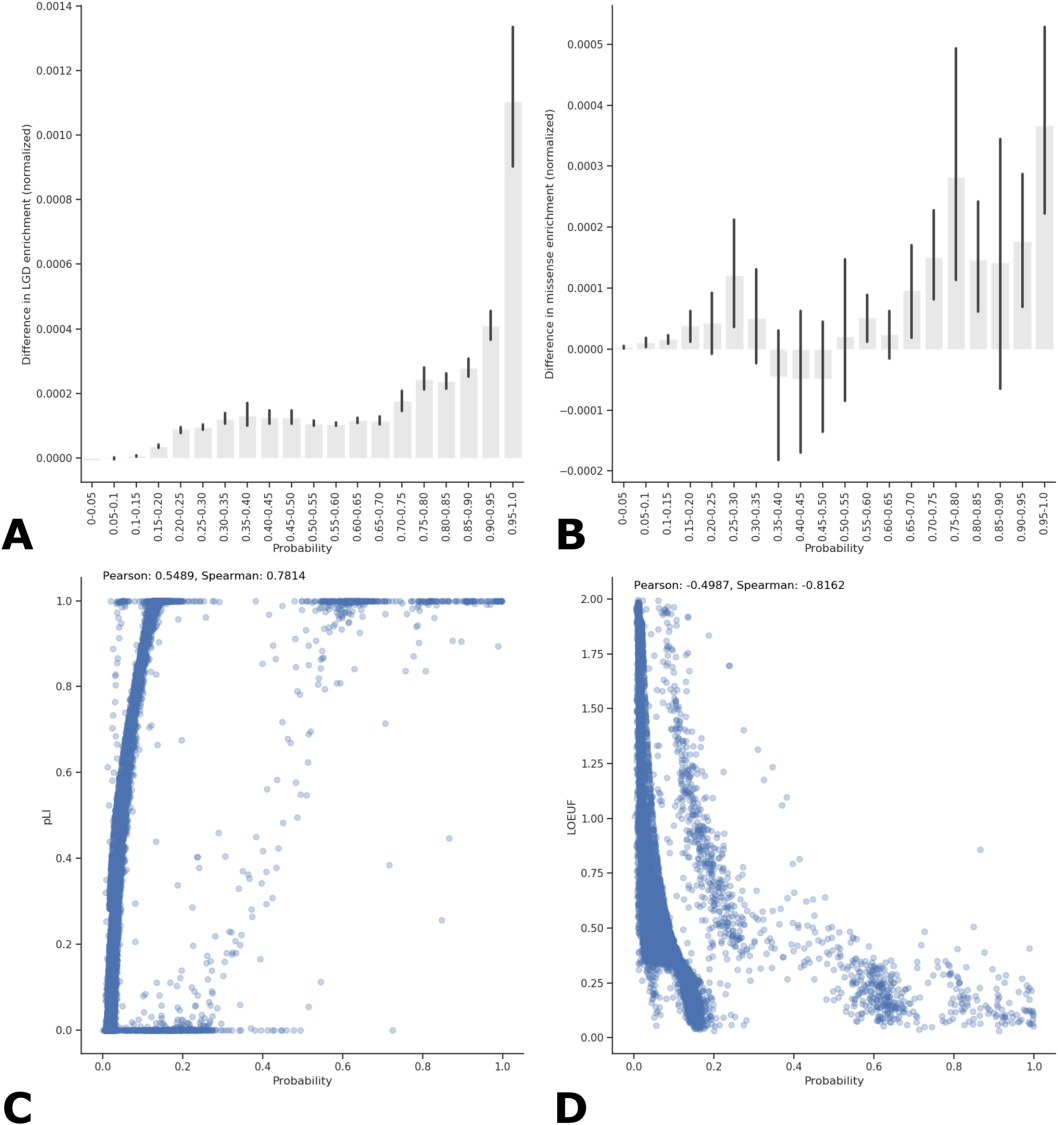
Increased enrichment of de novo LGD and missense mutation in NDD cases relative to unaffected controls in highly ranked NDD genes according to an SNN trained on an LGD-specific feature matrix. Applying a trained SNN on artificial samples containing a single unique LGD variant allows the SNN to rank genes according to their relative importance to NDD risk with respect to LGD coding variation. The difference in enrichment in NDD cases versus controls per ranked gene is calculated by Eq. 2 (Additional File 1) and displayed on the y-axes. Increasing probability (x-axes) indicates increasing importance to NDD risk. The average predicted probability was determined for each artificial sample over 100 independent iterations, and 95% CI are shown. At increasing probabilities for artificial samples with LGD variants, a steady, increased enrichment of LGD in cases (A) is observed, and a slight enrichment of missense variation (B) in cases relative to controls is also observed at increasing probabilities. The probability (ranks) assigned to genes is significantly correlated with both pLI (C) and LOEUF (D) values retrieved from gnomAD (v2.1.1). pLI values range from 0 to 1, where values above 0.9 suggest intolerance to LGD mutation, whereas LOEUF values represent a ratio of observed over expected LGD mutations and values less than 0.35 suggest intolerance to LGD mutation

For artificial LGD samples (that each possess a single LGD variant in a unique gene), an increased enrichment of LGD variants is observed in NDD cases relative to unaffected controls at increasing predicted probabilities (Fig. 3A), and a slight increased enrichment of missense variants is also observed in NDD cases for genes ranked according to a trained LGD-specific SNN (Fig. 3B). The difference in enrichment $({E}_{diff})$ of LGD or missense mutation in cases relative to controls per gene is calculated by Eq. 2 (Additional File 1). Significant correlation exists between pLI (*p-value* < 2.25e − 79) and LOEUF (*p-value* < 1.09e − 63) values with predicted probability ranks for artificial LGD samples (Fig. 3C, D). For gene rankings produced by a missense-specific SNN, similar trends in enrichment of de novo coding variation in NDD cases relative to controls are observed, although the range of probabilities predicted by the missense-specific SNN narrows compared to the LGD-specific SNN, and the strength of correlation amongst pLI and LOEUF values with predicted probabilities is reduced (Supplementary Fig. 5).

For the LGD-specific SNN model, inclusion of gene score features generated from pLI, LOEUF, RVIS, and PhastCons produces rankings with greater enrichment of LGD variation in cases relative to controls at higher probabilities than an LGD-specific SNN model trivially trained on one-hot encoded mutation information that excludes gene score features (Supplementary Fig. 6).

## Discussion

To distinguish NDD cases from unaffected controls at extremely low FPRs using de novo coding variation and measures of gene constraint and conservation, we developed a SNN with a customized objective function to maximize TP while simultaneously minimizing FP (Fig. 1). Although most cases of NDDs arise from a variety of classes of genetic variation, particularly common, non-coding, and structural variants, focusing specifically on de novo coding variation of relatively large effect size is a tradeoff to obtain significantly reduced FPR on a small but significant subset of samples. Compared to traditional machine learning techniques, such as RF, support vector machine (SVM), and LR (referred to as ‘baseline’ models), the constructed SNN is able to achieve greater TPR at FPR less than 0.01 given LGD-specific variation (Table 1, Fig. 2). The ability of the SNN to capture more than 30% of cases at FPR < 0.01, corresponding to at least 5.29% more cases than any baseline model (Table 1), indicates that the use of a SNN with the custom loss function (Eq. 1) is beneficial in classifying NDD cases at very low FPR. Note that it is estimated that LGD variants have been observed in roughly 10% of ASD cases and up to 30% of DD cases (Wang et al., 2021). Thus, our results indicate that the proposed SNN should be able to identify > 3% of ASD and > 10% of all DD cases while having an FPR of virtually zero simply by considering de novo LGD variants.

To demonstrate that gene scores related to constraint and conservation, including pLI, LOEUF, RVIS, and phastCons, were useful and necessary for the SNN to yield elevated TPR at FPR < 0.01 compared to baseline models given LGD-specific variation, the performance of trivially trained SNN and baseline models were measured (Supplementary Table 3, Supplementary Fig. 2). During trivial training, *only* a feature matrix of one-hot encoded values (1 or 0) denoting the presence or absence of a de novo coding variation within a gene were provided as input features to models. We note that most de novo mutations retrieved from denovo-db were identified via simplex studies that facilitate the identification of de novo variants, thus potentially introducing biased prediction in favor of variants identified via simplex rather than multiplex studies. We would also like to note that multiplex NDD cases will have a potentially lower chance of being caused by de novo variants and thus reduce the ability of our model’s accurate prediction of these cases. Similar TPR at FPR < 0.01 values are reported for trivially trained and trivially trained baseline models, indicating that the inclusion of gene score features greatly contributes to the SNN's improved ability to classify NDD cases at very low FPR.

In addition, a simple ensemble method that uses the average predicted probability from SNN and baseline model predictions is able to identify NDD cases at greater TPR at FPR < 0.01 and slightly increased precision at lowered recall than any of its constituent models (Table 1, Supplementary Fig. 4). Excluding SNN predictions from the ensemble model reveals that the SNN, compared to baseline models, contributes substantially to the ensemble model’s ability to accurately classify NDD cases at low FPR values. In fact, for LGD-specific variation, an ensemble method that excludes SNN predictions produces decreased TPR at FPR < 0.01 metrics compared to the SNN alone (Table 1).

The ability of SNN and baseline models to use only missense variation to identify NDD cases is relatively poor. However, the incorporation of both missense and LGD-specific predictions during ‘combined’ prediction for samples containing both LGD and missense variation, in which the maximum predicted probability from two separately trained missense- and LGD-specific models are returned, increases average TPR at FPR < 0.01 compared to using only probabilities predicted by an LGD-specific model (Table 1, Fig. 2). The improved performance of combined predictions indicates that certain samples possessing very deleterious missense variation (in addition to LGD variation) are correctly classified as cases when the predicted probability associated with the missense-specific model, rather than the LGD-specific model, is retrieved.

SNNs trained on LGD- and missense-specific feature matrices containing de novo coding variation from NDD cases and controls are able to rank genes according to their relative importance to NDD risk when applied to artificial samples which each contain a single type of de novo variant in a single gene (Supplementary Table 6). An increased enrichment of de novo LGD and missense mutation in NDD cases relative to controls is observed in highly ranked genes (those with higher predicted probability of being a case) using LGD-specific variation (Fig. 3). Significant, strong correlation exists between predicted probability for artificial samples for both the pLI and LOEUF constraint metrics, showing that the ranking via LGD-specific variation can accurately detect most high risk NDD genes. Among the 50 most highly ranked genes using LGD-specific variation, a total of 47 out of 50 genes are classified as high confidence (39 genes with score 1), strong candidate (6 genes with score 2), and suggestive evidence (2 genes with score 3) autism spectrum disorder (ASD) risk genes, including genes relevant to syndromes, according to SFARI Gene and OMIM (Supplementary Table 6). Among genes with predicted probabilities greater than 0.90 (ranks 1–55), four genes (*WDR45*, *CLTC*, *BRPF1*, and *GATAD2B*) do not possess SFARI annotations, but have been associated with neurodegeneration and intellectual disability according to OMIM annotations. Highly ranked genes lacking both SFARI Gene scores and OMIM annotations suggest candidate NDD genes susceptible to de novo LGD variation. Evidence of association with NDDs [*ZFHX3* (Fuller et al., 2018), *CHD5* (Parenti et al., 2021)*, UBR3* (Murcia Pienkowski et al., 2020)) or enrichment of de novo LGD mutation in NDD cases (*ANP32A, SKIDA1* (Coe et al., 2019)), neurodegeneration (*ANP32A* (Podvin et al., 2020), *HECTD1* (Schmidt et al., 2021)), gliomas (*LARP4B* (Koso et al., 2016)), synapses and neuronal formation (*LMTK3* (Takahashi et al., 2020), *DOT1L* (Franz et al., 2019)] have been studied in model organisms, cell lines, and families for these candidate NDD genes.

Weaker correlation is observed for missense-specific rankings with pLI and LOEUF values, and enrichment of de novo non-synonymous mutation is also present in NDD cases relative to controls, although to a lesser extent compared to LGD-specific rankings (Supplementary Fig. 5). The missense-specific rankings are distinct from LGD rankings in their ability to identify genes potentially sensitive to missense variation (Supplementary Table 6). Among highly ranked genes lacking SFARI Gene scores and OMIM annotations, previous studies suggest association with NDDs and schizophrenia [*OBSCN* (Hashimoto et al., 2016), *PLEC* (Dincer et al., 2015), *RYR2* (Lieve et al., 2019), *ZSWIM8* (Tischfield et al., 2017)], cortical formation and thickness [*LAMA5* (Omar et al., 2017), *GOLGA3* (Kim et al., 2017)), and neurodegenerative diseases (*PKHD1* (Santos-Laso et al., 2020), *DNAH*1 (Thonberg et al., 2017)].

Our results indicate that we can accurately predict a small, yet significant fraction of NDD cases using de novo coding variants. Currently, whole-exome or whole-genome sequencing of trios is not common practice. However, to make the early prediction of these disorders a reality, such sequencing should become common practice. Furthermore, our approach only covers a small fraction of affected patients and additional methods that use other types of biomolecular signatures, such as common variants, rare non-coding variants, and epigenomic markers, are needed to increase the reach of early prediction to a larger fraction of cases.

## Conclusions

In summary, the described SNN identifies NDD cases at higher TPR while having very low FPR in comparison to traditional machine learning methods. Several factors contribute to the improved performance of the proposed approach, namely: the use of gene constraint and conservation features in LGD-specific prediction and a custom loss function that specifically seeks to maximize the TPR while minimizing the FPR. An ensemble method, aggregated from SNN and baseline model predictions, is able to correctly classify a greater proportion of cases at FPR < 0.01 compared to any individual model. The SNN itself is a major contributor to increased TPR at FPR < 0.01 observed in the ensemble model. Although de novo missense mutation alone is a poor predictor of case status relative to LGD mutation, missense-specific predictions are useful during combined prediction for identifying additional cases that possess highly deleterious missense mutation in addition to LGD mutation. Fully trained SNNs on LGD- or missense-specific variation are also useful in NDD risk gene prioritization, revealing candidate NDD genes enriched in de novo non-synonymous mutations in NDD cases relative to controls.

## Supplementary Information

Below is the link to the electronic supplementary material.

Supplementary file1 (PDF 1948 KB) Additional File 1 Includes six figures (Supplementary Fig. 1–6), five tables (Supplementary Tables 1–5), and supplementary methods

Supplementary file2 (XLSX 2084 KB) Supplementary Table 6 (XLS). Ranking indicating relative importance of a gene to NDD risk according to rare de novo LGD and missense coding variation. Ranks, also known as the predicted probability of being a case for an artificial sample, closer to 1 symbolize greater importance to NDD risk. Multiple rankings are shown based on input features provided to SNN prediction models. Rankings are displayed on separate tabs, in which a tab label beginning with 'LGD' and 'Missense' indicates rankings based on LGD- and missense-specific variation, respectively. Tab labels containing 'Trivial' correspond to rankings created using trivial one-hot encoding of de novo mutations in input feature matrices, whereas labels containing 'Final' correspond to non-trivially trained models, in which LGD-specific models use both mutation information and gene score features. SFARI Gene scores (‘score’) and syndromic status (‘syndromic’) and OMIM disease associations (‘OMIM’) are also displayed

## Data Availability

Codes associated with the SNN and analysis are available at https://github.com/jchow32/EarlyPredictionSNN.

## Abbreviations

ASD: Autism spectrum disorder
AUC: Area under the curve
CI: Confidence interval
DD: Developmental disability
FPR: False positive rate
ID: Intellectual disability
LGD: Likely gene-disruptive
LOEUF: Loss-of-function observed/expected upper bound fraction
NDD: Neurodevelopmental disorder
pLI: Probability of loss-of-function intolerance
PR-AUC: Precision recall-area under the curve
ROC-AUC: Receiver operating characteristic-area under the curve
RVIS: Residual Variation Intolerance
SSC: Simons Simplex Collection
SNN: Shallow neural network
SVM: Support-vector machine
TPR: True positive rate
