# Supplementary material for: Prediction of Neurodevelopmental Disorders Based on De Novo Coding Variation
Source: J Autism Dev Disord. 2022 May 20;53(3):963–76. doi: 10.1007/s10803-022-05586-z (PMC9986216; doi:10.1007/s10803-022-05586-z)
Supplement: Supplementary file 1 — Supplementary file1 (PDF 1948 KB) Additional File 1 Includes six figures (Supplementary Fig. 1–6), five tables (Supplementary Tables 1–5), and supplementary methods [file 10803_2022_5586_MOESM1_ESM.pdf]

# Prediction of neurodevelopmental disorders based on *de novo* coding variation

Julie C. Chow,[1]* Fereydoun Hormozdiari[2]*

[1]UC Davis Genome Center, University of California, Davis, Davis, CA 95616, USA

[2]UC Davis Genome Center, University of California, Davis, Davis, CA 95616, USA; MIND Institute, University of California, Davis, 95817 USA; Biochemistry and Molecular Medicine, University of California, Davis, 95616 USA

*Correspondence: jcchow@ucdavis.edu, fhormozd@ucdavis.edu

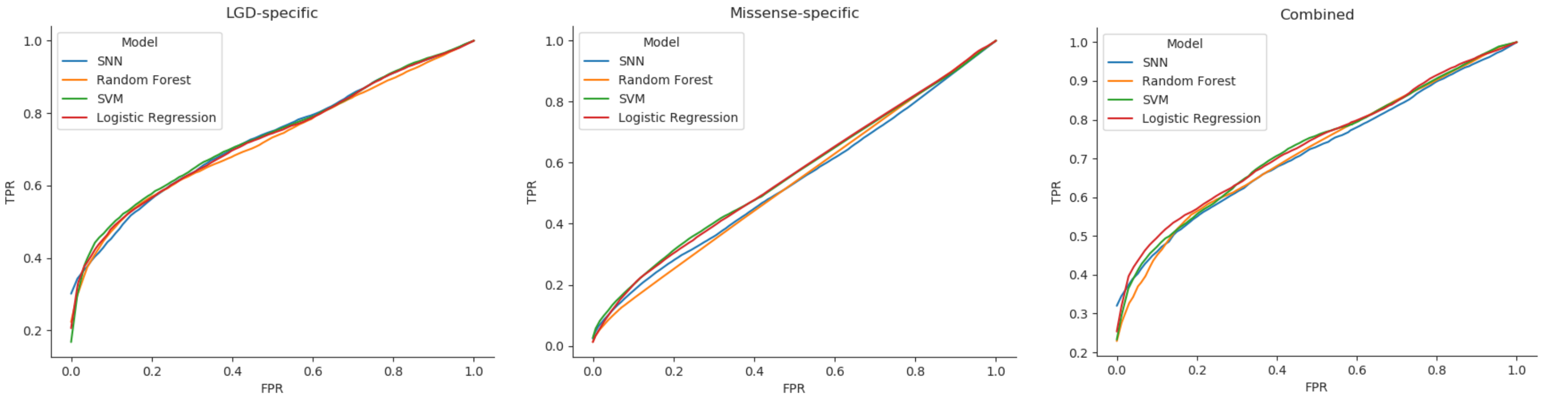


**Supplementary Figure 1**. Receiver operating characteristic (ROC) curves for LGD- and missense-specific shallow neural net (SNN) and baseline (random forest, SVM, and logistic regression) models at all false positive rate (FPR) thresholds.

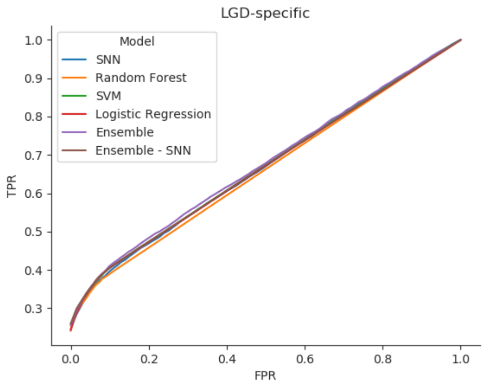
LGD-specific
Model
SNN
Random Forest
SVM
Logistic Regression
Ensemble
Ensemble - SNN
TPR
FPR

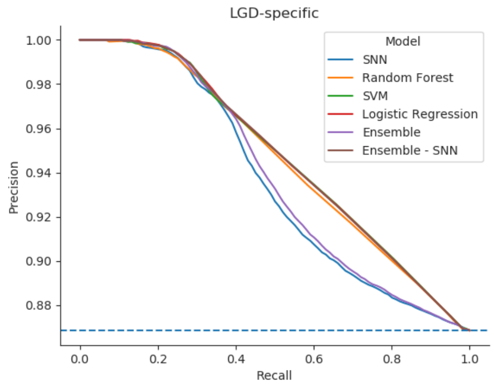
LGD-specific
Model
SNN
Random Forest
SVM
Logistic Regression
Ensemble
Ensemble - SNN
Precision
Recall

**A**

**B**

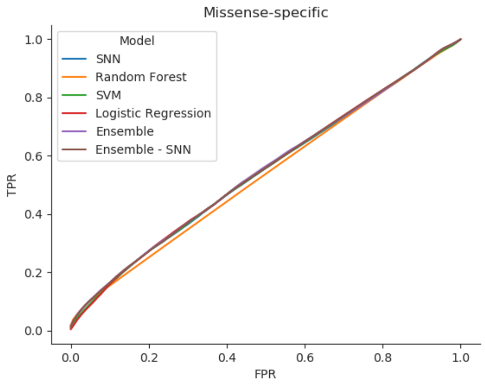
Missense-specific
Model
SNN
Random Forest
SVM
Logistic Regression
Ensemble
Ensemble - SNN
TPR
FPR

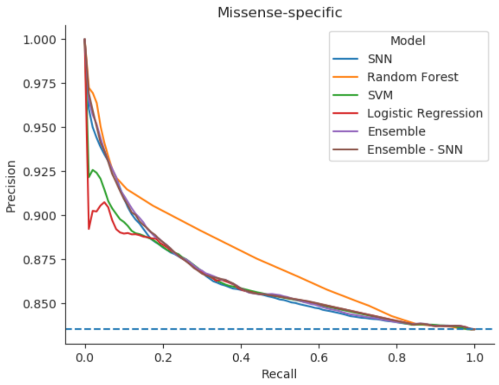
Missense-specific
Model
SNN
Random Forest
SVM
Logistic Regression
Ensemble
Ensemble - SNN
Precision
Recall

**C**

**D**

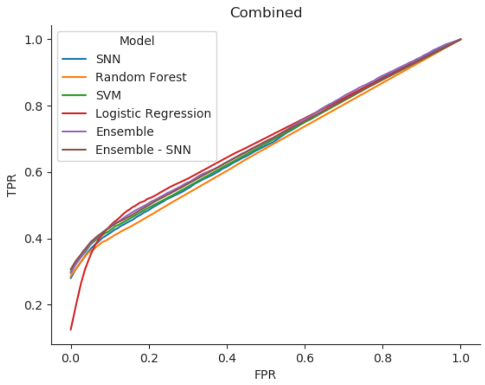
Combined
Model
SNN
Random Forest
SVM
Logistic Regression
Ensemble
Ensemble - SNN
TPR
FPR

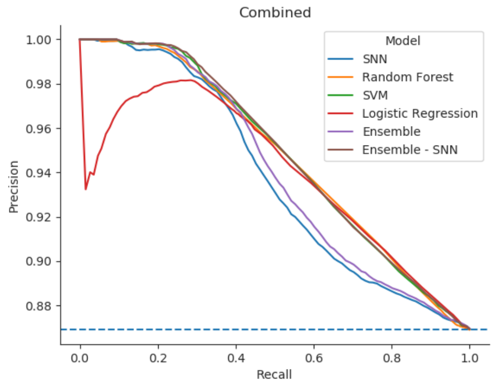
Combined
Model
SNN
Random Forest
SVM
Logistic Regression
Ensemble
Ensemble - SNN
Precision
Recall

**E**

**F**

**Supplementary Figure 2**. Receiver operating characteristic (ROC) and precision recall (PR) curves for LGD- and missense-specific SNN, baseline (random forest, SVM, and logistic regression), and ensemble models trivially trained on only one-hot encoded mutation information. 'Ensemble - SNN' refers to an ensemble model generated only from the predictions of baseline models. For a given sample, the ensemble model uses the average of the predicted probabilities from SNN and baseline models. SNN, baseline, and ensemble models perform similarly while trained only on LGD-specific (A-B), missense-specific (C-D), and combined mutation information (E-F). Models trained on missense-specific (C-D) variation alone are poor predictors of NDD status.

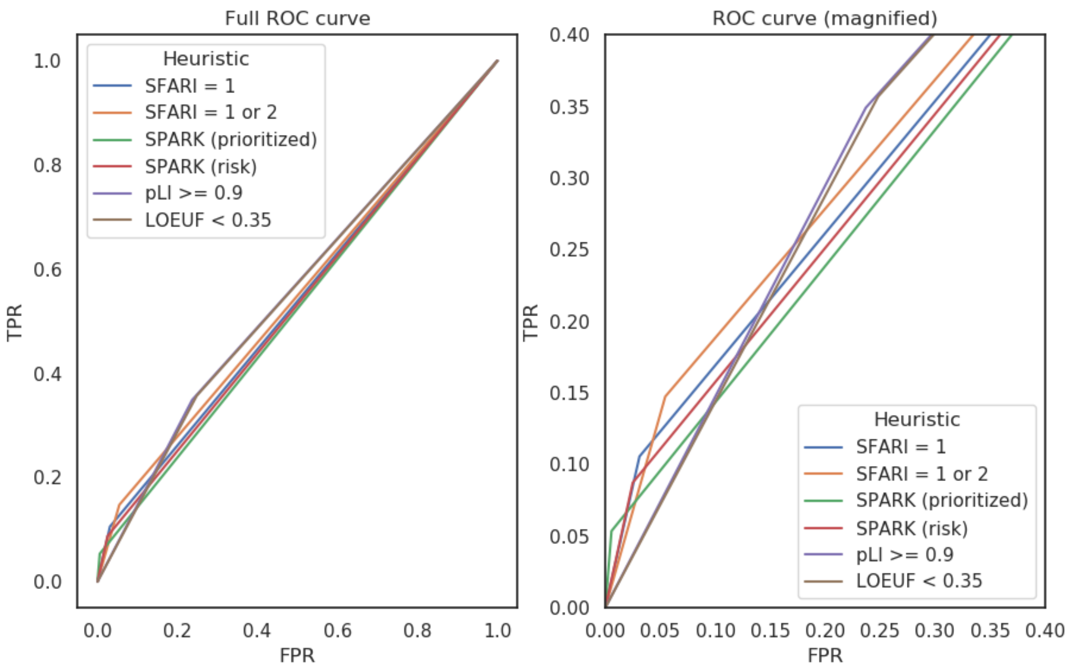


**Supplementary Figure 3.** Receiver operating characteristic (ROC) curves for three heuristics. For each heuristic, samples with a likely gene-disruptive (LGD) mutation in genes within a particular gene set were classified as cases. The full range of the ROC curve is displayed on the left, and a magnification is displayed on the right.

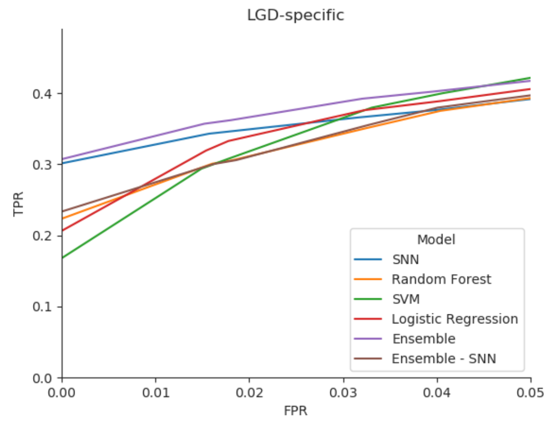
LGD-specific
TPR
FPR
Model
SNN
Random Forest
SVM
Logistic Regression
Ensemble
Ensemble - SNN

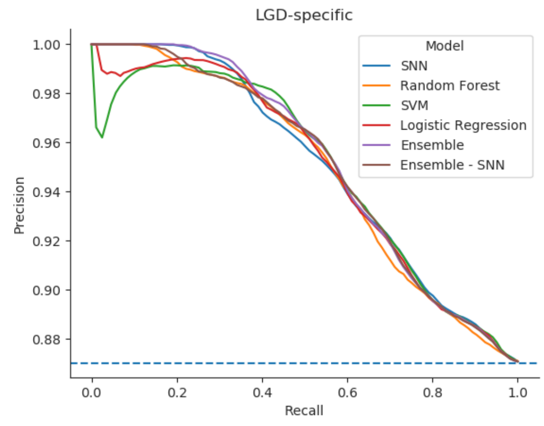
LGD-specific
Precision
Recall
Model
SNN
Random Forest
SVM
Logistic Regression
Ensemble
Ensemble - SNN

**A** **B**

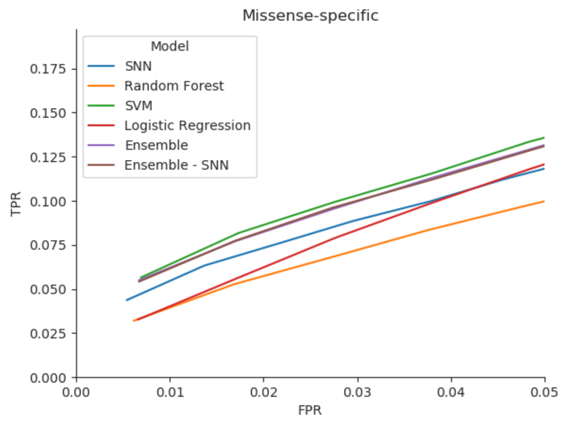
Missense-specific
TPR
FPR
Model
SNN
Random Forest
SVM
Logistic Regression
Ensemble
Ensemble - SNN

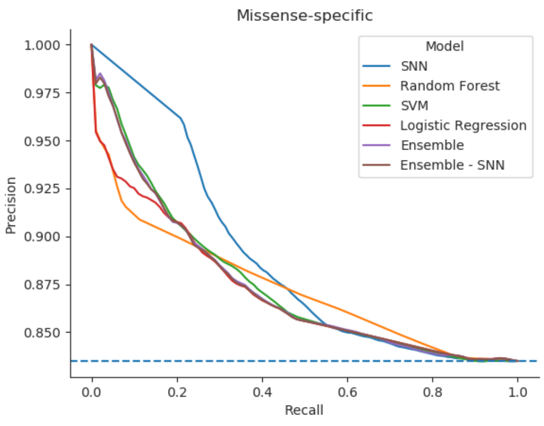
Missense-specific
Precision
Recall
Model
SNN
Random Forest
SVM
Logistic Regression
Ensemble
Ensemble - SNN

**C** **D**

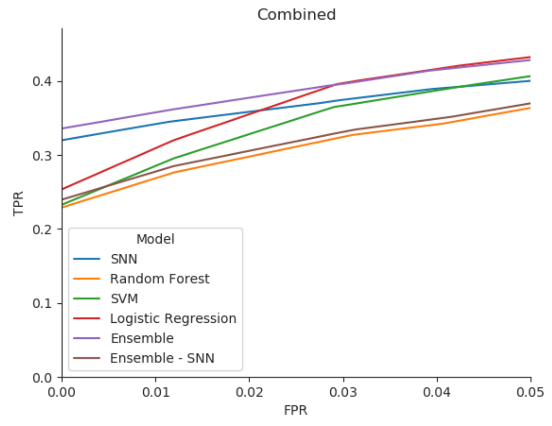
Combined
TPR
FPR
Model
SNN
Random Forest
SVM
Logistic Regression
Ensemble
Ensemble - SNN

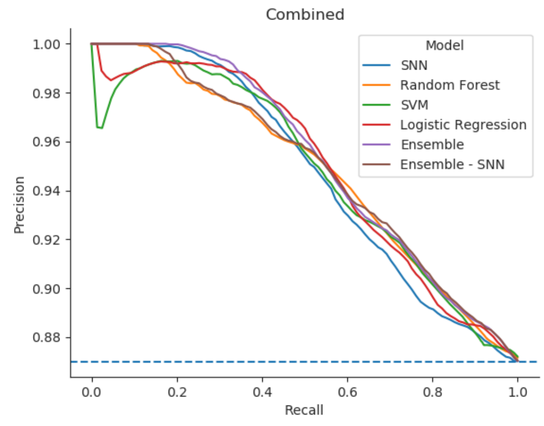
Combined
Precision
Recall
Model
SNN
Random Forest
SVM
Logistic Regression
Ensemble
Ensemble - SNN

**E** **F**

**Supplementary Figure 4**. Receiver operating characteristic (ROC) curves at low false positive rate (FPR) and precision recall (PR) curves for LGD- and missense-specific SNN, baseline (random forest, SVM, and logistic regression), and ensemble models. Models trained on LGD-specific variation feature matrices additionally use constraint and conservation gene score information, whereas models provided with missense-specific feature matrices do not use gene score information. For a given sample, the ensemble model uses the average of the predicted probabilities from SNN and baseline models. Ensemble - SNN refers to an ensemble of baseline models while excluding SNN predictions. A-B) For LGD-specific features, the ensemble model and SNN achieve greater TPR at low FPR < 0.01 compared to baseline models, a trend which is evident even at FPR < 0.05. Increased precision at low recall is observed for an ensemble model trained on LGD-specific variation. C-D) Models trained on missense-specific variation alone are poor predictors of NDD status; SNN and baseline models show similar TPR at FPR < 0.05, with the SNN and ensemble models achieving slightly TPR higher rates at low FPR. All models display comparable precision at low recall. E-F) For combined prediction for samples with both missense and LGD variation, the proportion of cases captured at FPR < 0.01 is largest for the ensemble model, followed by the SNN. The ensemble model achieves the largest precision at low recall thresholds.

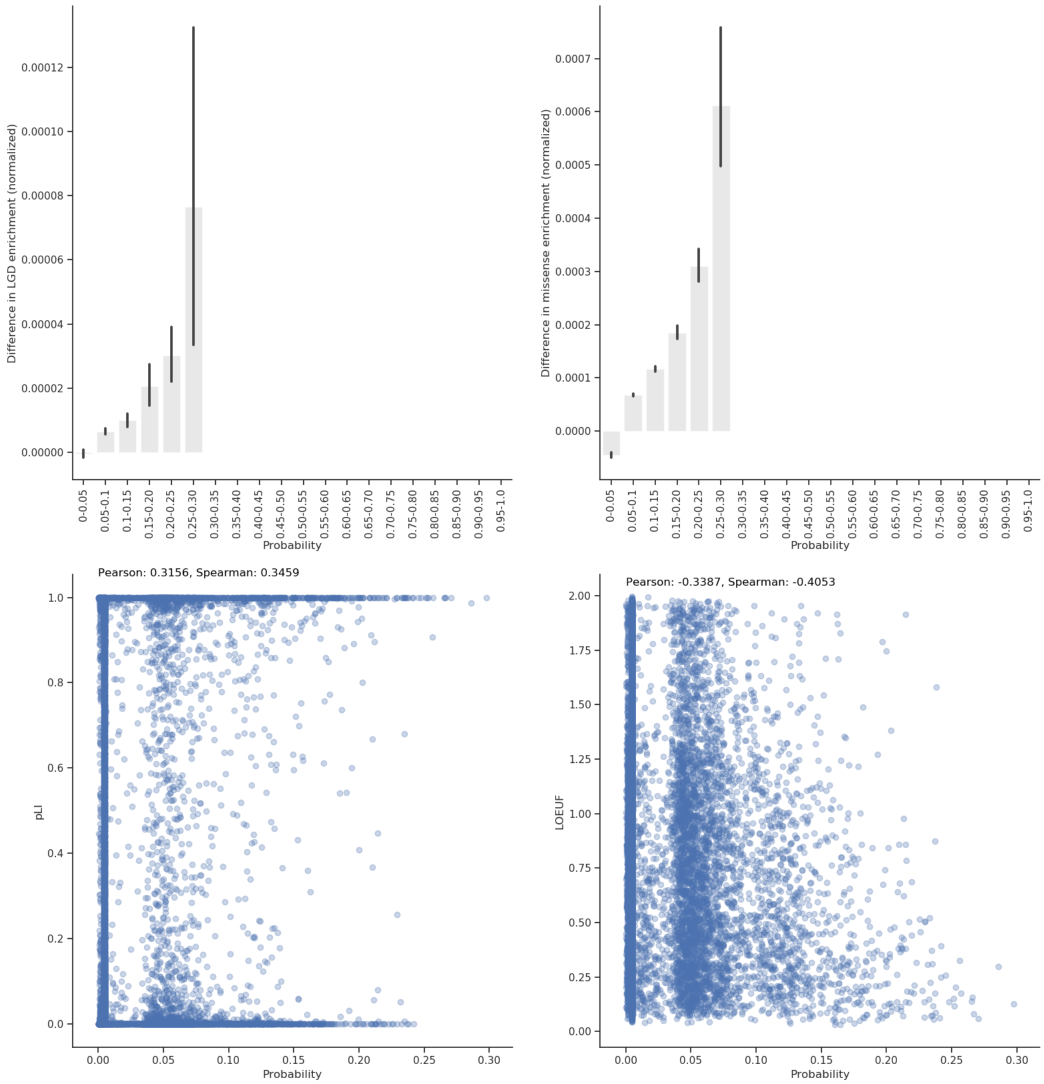


**Supplementary Figure 5**. Increased enrichment of *de novo* LGD and missense mutation in NDD cases relative to unaffected controls in more highly ranked NDD genes according to an SNN trained on an missense-specific feature matrix. Applying a trained SNN on artificial samples containing a single unique missense variant allows the SNN to rank genes according to their relative importance to NDD risk with respect to missense coding variation. The difference in enrichment in NDD cases versus controls is

calculated by **Equation 2** (**supplementary methods**) and displayed on the y-axes. Increasing probability (x-axes) indicates increasing importance to NDD risk. The average predicted probability was determined for each artificial sample over 100 independent iterations, and 95% confidence intervals are shown. At increasing probabilities for artificial samples with missense variants, an increased enrichment of LGD in cases (A) and missense in cases (B) is observed. The probability (ranks) assigned to genes is weakly correlated with both pLI (C) and LOEUF (D) values.

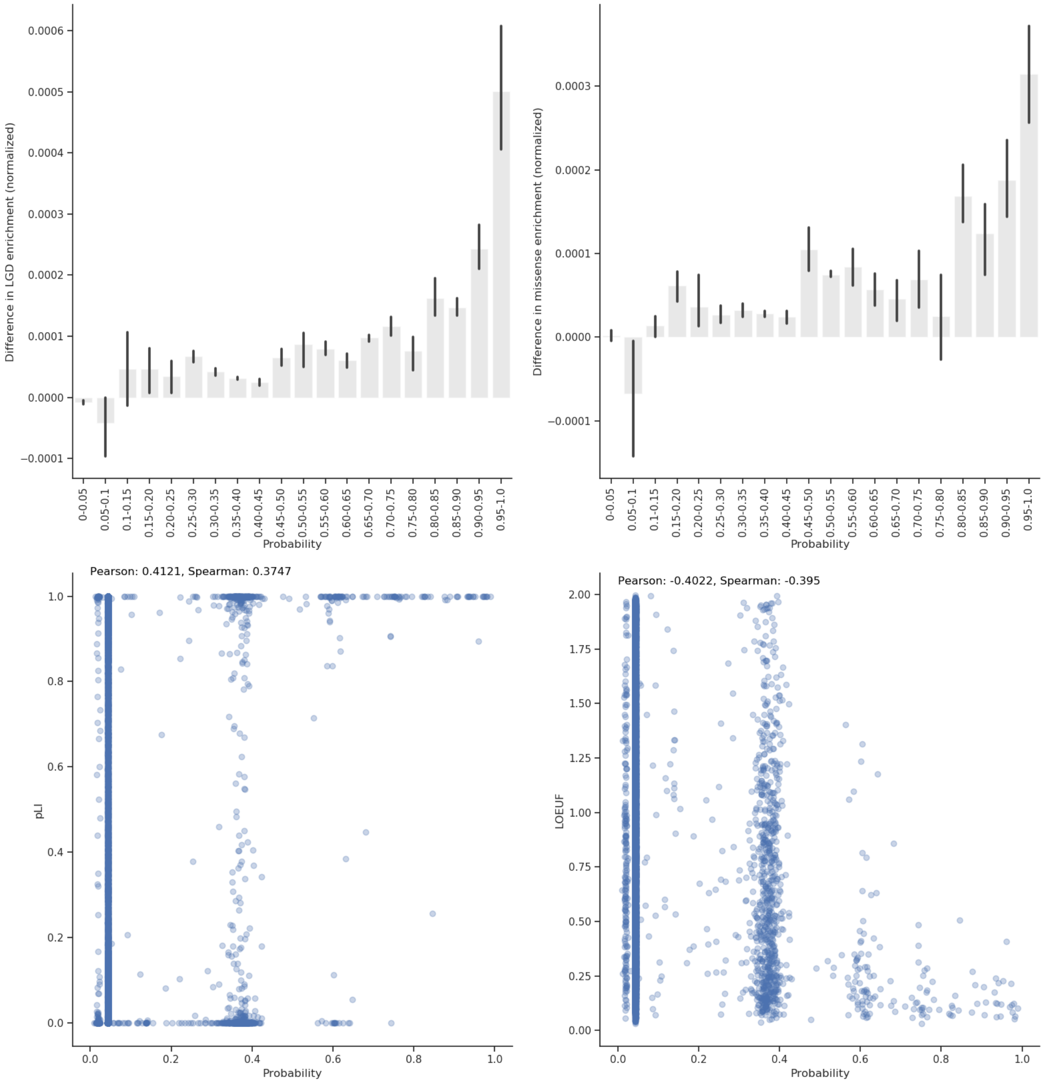


**Supplementary Figure 6.** Increased enrichment of *de novo* LGD and missense mutation in NDD cases relative to unaffected controls in highly ranked NDD genes according to an SNN trivially trained LGD-specific feature matrix consisting only of one-hot encoded mutation information. Applying a trivially trained SNN on artificial samples containing a single unique LGD variant allows the SNN to rank genes according to their relative importance to NDD risk with respect to LGD coding variation. The difference in

enrichment in NDD cases versus controls is calculated by **Equation 2** and displayed on the y-axes. Increasing probability (x-axes) indicates increasing importance to NDD risk. The average predicted probability was determined for each artificial sample over 100 independent iterations, and 95% confidence intervals are shown. At increasing probabilities for artificial samples with LGD variants, a steady, increased enrichment of LGD in cases (A) is observed. A slight enrichment of missense variation (B) in cases relative to controls is also observed at larger probabilities. The probability (ranks) assigned to genes is weakly correlated with both pLI (C) and LOEUF (D) values.

**Supplementary Table 1.** Neurodevelopmental disorder samples retrieved from denovo-db and associated primary phenotypes.

| Study | Primary Phenotype | Cases | Controls |
|---|---|---|---|
| Simons Simplex Collection | Autism | 2,508 | 1,911 |
| ASC | Autism | 1,445 | |
| MSSNG | Autism | 1,625 | |
| NIMH | Autism | 10 | |
| Hashimoto | Autism | 30 | |
| GoNL | Control | | 250 |
| Gulsuner | Control | | 84 |
| Rauch | Intellectual disability | 51 | |
| DDD | Developmental disorder | 4,293 | |

**Supplementary Table 2**. Search space for optimal hyperparameters. A single parameter is varied while other values are held constant on values most frequently determined to yield the highest average true positive rate (TPR) at false positive rate (FPR) < 0.01 in 100 independent iterations.

| Batch size | $\lambda_1$ | $\lambda_2$ | Neurons |
|---|---|---|---|
| [8, 16, 32] | 100 | 1e-5 | 16 |
| 32 | [70, …, 120] | 1e-5 | 16 |
| 32 | 100 | [1e-6, …, 1e-2] | 16 |
| 32 | 100 | 1e-5 | [8, 16, 32] |

**Supplementary Table 3.** Average true positive rate (TPR) at false positive rate (FPR) < 0.01, area under the curve (ROC-AUC), and precision recall area under the curve (PR-AUC) for LGD-specific, missense-specific, and combined shallow neural net (SNN), baseline, randomized predictions, and ensemble models trivially trained on feature matrices containing only one-hot encoded mutation information. 'Ensemble - SNN' refers to an ensemble model generated only from the predictions of baseline models. Average performance metrics are measured over 100 independent iterations of randomized training/testing splits on the testing set, in which the same training/testing partition is provided to all models at each iteration. Confidence intervals (95% CI) are indicated in parentheses, followed by a z-score quantifying the deviance from the mean performance metric of a certain model and the randomized model (**supplementary methods**).

| Input features | Model | TPR at FPR < 0.01 (95% CI); z-score | ROC-AUC (95% CI); z-score | PR-AUC (95% CI); z-score |
|---|---|---|---|---|
| LGD-specific | SNN | 0.24532 (0.2364, 0.2544); 3.85542 | 0.66696 (0.6616, 0.6727); 2.84528 | 0.93597 (0.9344, 0.9377); 4.39382 |
| | Random forest | 0.24593 (0.2358, 0.2559); 3.86630 | 0.66027 (0.657, 0.6636); 3.13763 | 0.94557 (0.9440, 0.9469); 5.40379 |
| | SVM | 0.25911 (0.2504, 0.2676); 4.43549 | 0.67015 (0.6668, 0.6734); 3.33195 | 0.94637 (0.9448, 0.9478); 5.37662 |
| | Logistic regression | 0.24141 (0.234, 0.2487); 4.71332 | 0.66768 (0.6644, 0.6711); 3.28010 | 0.94670 (0.9452, 0.9482); 5.51134 |
| | Ensemble | 0.25526 (0.2463, 0.2645); 4.45173 | 0.67449 (0.6697, 0.6794); 3.05424 | 0.93795 (0.9362, 0.9395); 4.59742 |
| | Ensemble - SNN | 0.25909 (0.2503, 0.2672); 4.38812 | 0.66892 (0.6654, 0.6725); 3.30916 | 0.94658 (0.9451, 0.948); 5.46046 |
| | Randomized | 0.01590 (0.0133, 0.019) | 0.51564 (0.5086, 0.5233) | 0.8684 |
| Missense-specific | SNN | 0.01274 (0.011, 0.0146); 0.81483 | 0.54538 (0.5427, 0.548); 1.81664 | 0.86321 (0.8621, 0.8643); 4.20065 |
| | Random forest | 0.01788 (0.0157, 0.0205); 0.94788 | 0.53337 (0.5311, 0.5357); 1.39329 | 0.87390 (0.8723, 0.8757); 3.85907 |
| | SVM | 0.00777 (0.0064, 0.0093); 0.41541 | 0.54479 (0.5424, 0.5472); 1.86765 | 0.86141 (0.8723, 0.8757); 3.40463 |
| | Logistic regression | 0.00442 (0.0037, 0.0052); 0.09893 | 0.54581 (0.5435, 0.5484); 1.91177 | 0.86023 (0.8602, 0.8626); 3.57777 |
| | Ensemble | 0.01385 (0.0121, 0.0157); 0.87097 | 0.54842 (0.5458, 0.551); 1.96634 | 0.86460 (0.8591, 0.8613); 4.51338 |

| | | | | |
|---|---|---|---|---|
| | Ensemble - SNN | 0.01385 (0.0123, 0.0158); 0.87638 | 0.54726 (0.545, 0.5496); 1.98008 | 0.86464 (0.8636, 0.8656); 4.48374 |
| | Randomized | 0.00382 (0.0031, 0.0046) | 0.49954 (0.496, 0.503) | 0.8353 |
| Combined | SNN | 0.27952 (0.2671, 0.2929); 2.93042 | 0.67796 (0.6705, 0.6855); 2.45043 | 0.93764 (0.9356, 0.94); 3.17344 |
| | Random forest | 0.28581 (0.2743, 0.2972); 3.19354 | 0.66765 (0.6634, 0.6715); 2.74962 | 0.94663 (0.9449, 0.9484); 3.82637 |
| | SVM | 0.29840 (0.2867, 0.309); 3.43782 | 0.68337 (0.6785, 0.688); 2.89963 | 0.94854 (0.9466, 0.9506); 3.92888 |
| | Logistic regression | 0.12399 (0.1013, 0.1488); 0.63492 | 0.69015 (0.6841, 0.6963); 2.87051 | 0.93856 (0.9357, 0.9414); 2.92455 |
| | Ensemble | 0.29459 (0.2828, 0.306); 3.34720 | 0.68981 (0.6825, 0.6968); 2.70058 | 0.94080 (0.9387, 0.943); 3.42898 |
| | Ensemble - SNN | 0.30602 (0.2944, 0.3179); 3.49605 | 0.68776 (0.6831, 0.6925); 2.99052 | 0.94837 (0.9466, 0.9503); 3.91210 |
| | Randomized | 0.03293 (0.0274, 0.0387) | 0.51969 (0.512, 0.5281) | 0.8690 |

**Supplementary Table 4.** True positive (TPR) and false positive rates (FPR) for three heuristics. A sample was classified as a case if the sample contained a likely gene-disruptive (LGD) mutation in a set of risk genes where the gene 1) i) has a SFARI score of 1 or ii) a SFARI score of 1 or 2, 2) was classified as a SPARK i) prioritized gene or ii) risk gene, and 3) i) pLI >= 0.90 or ii) LOEUF < 0.35.

| Heuristic | TPR | FPR |
|---|---|---|
| SFARI, score 1 | 0.1056 | 0.0311 |
| SFARI, score 1 or 2 | 0.1474 | 0.0545 |
| SPARK (prioritized) | 0.0535 | 0.0056 |
| SPARK (risk) | 0.0873 | 0.025 |
| pLI >= 0.9 | 0.3491 | 0.2369 |
| LOEUF < 0.35 | 0.3569 | 0.2481 |

**Supplementary Table 5.** Average true positive rate (TPR) at false positive rate (FPR) < 0.01, area under the curve (ROC-AUC), and precision recall area under the curve (PR-AUC) for missense-specific shallow neural net (SNN), baseline, randomized predictions, and ensemble models using feature matrices containing only one-hot encoded deleterious (PrimateAI score >= 0.803) missense variation i) during training without removing any samples from the dataset or ii) during both training and testing by removing samples without deleterious missense variation from the dataset. 'Ensemble - SNN' refers to an ensemble model generated only from the predictions of baseline models. Average performance metrics are measured over 100 independent iterations of randomized training/testing splits on the testing set, in which the same training/testing partition is provided to all models at each iteration. Confidence intervals (95% CI) are indicated in parentheses, followed by a z-score quantifying the deviance from the mean performance metric of a certain model and the randomized model (**supplementary methods**).

| Input features | Model | TPR at FPR < 0.01 (95% CI); z-score | ROC-AUC (95% CI); z-score | PR-AUC (95% CI); z-score |
|---|---|---|---|---|
| Missense-specific (i) | SNN | 0.02829 (0.0211, 0.0359); 1.33101 | 0.54744 (0.542, 0.5525); 1.97382 | 0.87008 (0.8681, 0.8722); 6.10794 |
| | Random forest | 0.02660 (0.0202, 0.0336); 1.38829 | 0.54222 (0.5374, 0.5474); 1.76958 | 0.87564 (0.8715, 0.8797); 3.85456 |
| | SVM | 0.02584 (0.0175, 0.0337); 1.04541 | 0.55408 (0.5489, 0.5595); 2.29522 | 0.87556 (0.8726, 0.8783); 5.52276 |
| | Logistic regression | 0.01033 (0.0062, 0.0151); 0.57026 | 0.55447 (0.5493, 0.5595); 2.30987 | 0.87337 (0.8701, 0.8767); 4.51765 |
| | Ensemble | 0.02883 (0.0218, 0.0356); 1.43505 | 0.55427 (0.549, 0.5594); 2.27145 | 0.87188 (0.8695, 0.8741); 6.24093 |
| | Ensemble - SNN | 0.03000 (0.023, 0.037); 1.44297 | 0.55441 (0.5494, 0.5596); 2.31924 | 0.87547 (0.8727, 0.8782); 5.59827 |
| | Randomized | 0.00323 (0.0022, 0.0044) | 0.50044 (0.4931, 0.5083) | 0.83492 (0.8342, 0.8357) |
| Missense-specific (ii) | SNN | 0.09378 (0.0719, 0.116); 1.51357 | 0.63740 (0.626, 0.6487); 3.07228 | 0.93743 (0.9345, 0.9403); 4.09638 |
| | Random forest | 0.08738 (0.0595, 0.1177); 1.07341 | 0.62847 (0.6178, 0.639); 2.89912 | 0.94014 (0.9371, 0.9431); 4.26020 |
| | SVM | 0.07504 (0.0503, 0.1034); 0.94629 | 0.63587 (0.6268, 0.6452); 3.23138 | 0.94094 (0.9377, 0.9442); 4.39978 |
| | Logistic regression | 0.05182 (0.0247, 0.0802); 0.56745 | 0.63210 (0.6209, 0.642); 3.03888 | 0.93585 (0.9318, 0.9399); 3.39856 |

| | | | | |
|---|---|---|---|---|
| | Ensemble | 0.08648 (0.0647, 0.1114); 1.37385 | 0.64388 (0.6332, 0.6529); 3.31811 | 0.93892 (0.9361, 0.9416); 4.47331 |
| | Ensemble - SNN | 0.08155 (0.0578, 0.1037); 1.17750 | 0.63327 (0.6235, 0.6425); 3.06469 | 0.94012 (0.9372, 0.9431); 4.47532 |
| | Randomized | 0.01051 (0.0062, 0.0154) | 0.50026 0.485, 0.5142) | 0.89386 (0.8908, 0.8969) |

**Supplementary methods**

*Construction of LGD- and missense-specific feature matrices*

*De novo* LGD and missense variants were retrieved from samples with autism spectrum disorder, developmental disability, or intellectual disability directly from denovo-db (version 1.6.1) (C Yuen et al. 2017; De Rubeis et al. 2014; Deciphering Developmental Disorders Study 2017; Genome of the Netherlands Consortium 2014; Gulsuner et al. 2013; Hashimoto et al. 2016; Iossifov et al. 2014; Krumm et al. 2015; Michaelson et al. 2012; B. J. O'Roak et al. 2014; Brian J. O'Roak et al. 2012; Rauch et al. 2012; Turner et al. 2016, 2017; Werling et al. 2018; Yuen et al. 2016).

For a given individual for a particular gene, the presence of an LGD variant was indicated in the feature matrix with a 1, the absence of any *de novo* variants as a 0, and the presence of a missense variant as the associated *PrimateAI* score (Sundaram et al. 2018). For example, for a sample possessing both LGD and missense variation, the presence of missense variation is simply denoted as a 0 in the LGD-specific matrix. In the case of multiple *de novo* variants existing in a single gene in a single sample, the mutation is recorded in the feature matrix as the larger of the scores. For the model trained on an LGD-specific feature matrix (LGD-specific model), gene score features related to pLI, LOEUF, RVIS, and phastCons values were generated by matrix multiplication with the LGD-specific feature matrix(Karczewski et al. 2020; Petrovski et al. 2013; Siepel et al. 2005). The gene scores features were concatenated with the LGD-specific feature matrix to yield a matrix of size (samples by (genes + 4)). The missense-specific feature matrix uses a simplified set of features of size (samples by genes), only indicating the presence of missense mutations in genes.

Following the splitting of all samples into training and testing sets, during model training, min-max scaling (from scikit-learn MinMaxScaler()) is applied to the training set, and the testing set is transformed with the applied scaler. Class weights were balanced according to sklearn.utils.class_weight.compute_class_weight (version 0.22.1).

*Hyperparameter optimization*

During hyperparameter optimization (**Figure 1B**), K-fold stratified cross-validation (K=3) is applied to the training set, in which the input set is split into K folds. K-1 folds are used as training folds, and a single fold is used as a validation fold. Over K iterations, a different fold is selected as the validation fold. Optimal hyperparameters for SNNs are selected from the following possible values: batch size={8, 16, 32}, $\lambda_1$= {70, 80, 90, 100, 110, 120}, L2 regularization $\lambda_2$={1e-2, 1e-3, 1e-4, 1e-5, 1e-6}, and number of neurons in the hidden layer={8, 16, 32}. To decrease compute time and the hyperparameter search space, selected combinations (**Supplementary Table 2**) are evaluated. For the SNNs, for every validation fold and potential set of optimal hyperparameters, the probability of a being a case is retrieved for every individual in the validation fold and the TPR at FPR < 0.01 is determined. 'TPR at FPR < 0.01' is calculated by first identifying the largest predicted probability associated with a control in a validation fold, followed by determining the fraction of cases in the validation fold with predicted probabilities greater than that of the control with the largest predicted probability, which is equivalent to an FPR of 0 and necessarily lower than 0.01To provide a more conservative estimate of the TPR at FPR equal to 0, we refer to this value as 'TPR at FPR < 0.01'. The optimal set of hyperparameters are defined as the set of hyperparameters for which the largest average TPR at FPR < 0.01 is achieved in the validation folds.

During hyperparameter optimization for baseline models, optimal hyperparameters are selected by minimizing the model's corresponding loss function value (random forest: Gini impurity, SVM: hinge loss, logistic regression: cross entropy). Optimal hyperparameters are selected among the following values for the baselines models: Random Forest: trees={100, 200, 300, 400, 500}, maximum depth={32, 36, 40, 44, 48, 52}; SVM: C={10, 1, 1e-2, 1e-3}; logistic regression: C={10,000, 1,000, 100, 10, 1}. C is inversely proportional to L2 regularization strength in both SVM and logistic regression models.

*Assessment of model performance*

The average performance of a model is assessed over 100 independent iterations in which the training and testing sets are randomly partitioned and optimal hyperparameters are selected per iteration. For each iteration, the performance metrics TPR at FPR < 0.01, ROC-AUC, and PR-AUC are determined

from the predicted probabilities of samples in the testing set. Averages of these performance metrics and bootstrapped 95% confidence intervals are reported for LGD-specific, missense-specific, and combined predictions for our SNN, three baseline models, an ensemble model, and an ensemble model excluding SNN predictions (**Table 1**). The full ensemble model, for every independent iteration, returns the average predicted probability using the predicted probabilities from the SNN and baseline models for every sample in the testing set. The TPR at FPR < 0.01, ROC-AUC, and PR-AUC are reported similarly for the ensemble model on the resultant average probabilities from the SNN and baseline models. For each model, bootstrap confidence intervals (95%) and z-scores were calculated for each performance metric. The z-score was calculated as the difference between the mean performance metric for a certain model and the randomized model divided by the square root of the sum of the variances.

To compare the performance of SNN, baseline, and ensemble models to randomized predictions, probabilities were randomly generated from a uniform distribution and assigned to samples. Average random PR-AUC values were calculated by dividing the number of cases in a testing set by the total number of samples within the testing set. Models that were 'trivially trained' refer to using one-hot encoded feature matrices indicating only the presence or absence (denoted as 1 or 0, respectively) of *de novo* LGD or missense mutation. TPR and FPR values were retrieved for three heuristics, where a sample was classified as a case if the sample possessed an LGD mutation in a gene that was identified: 1) as a high risk or strong candidate ASD gene according SFARI Gene scores (https://gene.sfari.org/database/gene-scoring/), 2) in the prioritized SPARK gene list (https://simonsfoundation.s3.amazonaws.com/share/SFARI/Prioritized%20SPARK%20Gene%20List_for%20distribution_27Apr21.xlsx), or 3) to have elevated intolerance to mutation (pLI >= 0.9, LOEUF < 0.35) (Karczewski et al. 2020).

*Assessing enrichment of de novo mutation in NDD cases for ranked NDD risk genes*

To determine if enrichment of LGD (or missense) in NDD cases relative to unaffected controls is observed in highly ranked NDD risk genes, the difference in enrichment among NDD cases and controls

($E_{diff}$) is calculated per gene by **Equation 2**. The total number of LGD (or missense) mutations observed in cases ($M_{cases}$) for a certain gene within the test set is divided by the number of NDD cases retrieved from denovo-db ($N_{cases} = 9{,}962$), and the total number of LGD (or missense) mutations observed in controls ($M_{controls}$) for that gene within the test set is divided by the number of controls ($N_{controls} = 2{,}245$).

$$E_{diff} = (M_{cases} / N_{cases}) - (M_{controls} / N_{controls}) \quad \textbf{[Equation 2]}$$

**Web resources**

denovo-db, https://denovo-db.gs.washington.edu

Genome Aggregation Database (gnomAD), https://gnomad.broadinstitute.org/downloads

Online Mendelian Inheritance in Man (OMIM), https://www.omim.org

phastCons, http://hgdownload.cse.ucsc.edu/goldenpath/hg19/phastCons100way/

Residual Variation Intolerance Score (RVIS), http://genic-intolerance.org/

Scikit-learn, https://scikit-learn.org/stable/

SFARI Gene, https://gene.sfari.org/

C Yuen, R. K., Merico, D., Bookman, M., L Howe, J., Thiruvahindrapuram, B., Patel, R. V., et al. (2017). Whole genome sequencing resource identifies 18 new candidate genes for autism spectrum disorder. Nature Neuroscience, 20(4), 602–611. https://doi.org/10.1038/nn.4524

De Rubeis, S., He, X., Goldberg, A. P., Poultney, C. S., Samocha, K., Cicek, A. E., et al. (2014). Synaptic, transcriptional, and chromatin genes disrupted in autism. Nature, 515(7526), 209–215. https://doi.org/10.1038/nature13772

Deciphering Developmental Disorders Study. (2017). Prevalence and architecture of de novo mutations in developmental disorders. Nature, 542(7642), 433–438. https://doi.org/10.1038/nature21062

Genome of the Netherlands Consortium. (2014). Whole-genome sequence variation, population structure and demographic history of the Dutch population. Nature Genetics, 46(8), 818–825. https://doi.org/10.1038/ng.3021

Gulsuner, S., Walsh, T., Watts, A. C., Lee, M. K., Thornton, A. M., Casadei, S., et al. (2013). Spatial and temporal mapping of de novo mutations in schizophrenia to a fetal prefrontal cortical network. Cell, 154(3), 518–529. https://doi.org/10.1016/j.cell.2013.06.049

Hashimoto, R., Nakazawa, T., Tsurusaki, Y., Yasuda, Y., Nagayasu, K., Matsumura, K., et al. (2016). Whole-exome sequencing and neurite outgrowth analysis in autism spectrum disorder. Journal of Human Genetics, 61(3), 199–206. https://doi.org/10.1038/jhg.2015.141

Iossifov, I., O'Roak, B. J., Sanders, S. J., Ronemus, M., Krumm, N., Levy, D., et al. (2014). The contribution of de novo coding mutations to autism spectrum disorder. Nature, 515(7526), 216–221. https://doi.org/10.1038/nature13908

Karczewski, K. J., Francioli, L. C., Tiao, G., Cummings, B. B., Alföldi, J., Wang, Q., et al. (2020). The mutational constraint spectrum quantified from variation in 141,456 humans. *Nature*, *581*(7809), 434–443. https://doi.org/10.1038/s41586-020-2308-7

Krumm, N., Turner, T. N., Baker, C., Vives, L., Mohajeri, K., Witherspoon, K., et al. (2015). Excess of rare, inherited truncating mutations in autism. Nature Genetics, 47(6), 582–588. https://doi.org/10.1038/ng.3303

Michaelson, J. J., Shi, Y., Gujral, M., Zheng, H., Malhotra, D., Jin, X., et al. (2012). Whole Genome Sequencing in Autism Identifies Hotspots for De Novo Germline Mutation. Cell, 151(7), 1431–1442. https://doi.org/10.1016/j.cell.2012.11.019

O'Roak, B. J., Stessman, H. A., Boyle, E. A., Witherspoon, K. T., Martin, B., Lee, C., et al. (2014). Recurrent de novo mutations implicate novel genes underlying simplex autism risk. Nature Communications, 5, 5595. https://doi.org/10.1038/ncomms6595

O'Roak, Brian J., Vives, L., Fu, W., Egertson, J. D., Stanaway, I. B., Phelps, I. G., et al. (2012). Multiplex targeted sequencing identifies recurrently mutated genes in autism spectrum disorders.

Science (New York, N.Y.), 338(6114), 1619–1622. https://doi.org/10.1126/science.1227764

Petrovski, S., Wang, Q., Heinzen, E. L., Allen, A. S., & Goldstein, D. B. (2013). Genic Intolerance to Functional Variation and the Interpretation of Personal Genomes. *PLOS Genetics*, *9*(8), e1003709. https://doi.org/10.1371/journal.pgen.1003709

Rauch, A., Wieczorek, D., Graf, E., Wieland, T., Endele, S., Schwarzmayr, T., et al. (2012). Range of genetic mutations associated with severe non-syndromic sporadic intellectual disability: an exome sequencing study. Lancet (London, England), 380(9854), 1674–1682. https://doi.org/10.1016/S0140-6736(12)61480-9

Siepel, A., Bejerano, G., Pedersen, J. S., Hinrichs, A. S., Hou, M., Rosenbloom, K., et al. (2005). Evolutionarily conserved elements in vertebrate, insect, worm, and yeast genomes. *Genome Research*, *15*(8), 1034–1050. https://doi.org/10.1101/gr.3715005

Sundaram, L., Gao, H., Padigepati, S. R., McRae, J. F., Li, Y., Kosmicki, J. A., et al. (2018). Predicting the clinical impact of human mutation with deep neural networks. *Nature Genetics*, *50*(8), 1161–1170. https://doi.org/10.1038/s41588-018-0167-z

Turner, T. N., Coe, B. P., Dickel, D. E., Hoekzema, K., Nelson, B. J., Zody, M. C., et al. (2017). Genomic Patterns of De Novo Mutation in Simplex Autism. Cell, 171(3), 710-722.e12. https://doi.org/10.1016/j.cell.2017.08.047

Turner, T. N., Hormozdiari, F., Duyzend, M. H., McClymont, S. A., Hook, P. W., Iossifov, I., et al. (2016). Genome Sequencing of Autism-Affected Families Reveals Disruption of Putative Noncoding Regulatory DNA. American Journal of Human Genetics, 98(1), 58–74. https://doi.org/10.1016/j.ajhg.2015.11.023

Werling, D. M., Brand, H., An, J.-Y., Stone, M. R., Zhu, L., Glessner, J. T., et al. (2018). An analytical framework for whole genome sequence association studies and its implications for autism spectrum disorder. Nature genetics, 50(5), 727–736. https://doi.org/10.1038/s41588-018-0107-y

Yuen, R. K. C., Merico, D., Cao, H., Pellecchia, G., Alipanahi, B., Thiruvahindrapuram, B., et al. (2016). Genome-wide characteristics of de novo mutations in autism. NPJ genomic medicine, 1, 160271–

1602710. https://doi.org/10.1038/npjgenmed.2016.27
